# Longitudinal study on transmission of MRSA CC398 within pig herds

**DOI:** 10.1186/1746-6148-8-58

**Published:** 2012-05-18

**Authors:** Els M Broens, Carmen Espinosa-Gongora, Elisabeth AM Graat, Nadia Vendrig, Peter J Van Der Wolf, Luca Guardabassi, Patrick Butaye, Jens Peter Nielsen, Mart CM De Jong, Arjen W Van De Giessen

**Affiliations:** 1 Quantitative Veterinary Epidemiology Group, Wageningen Institute of Animal Sciences, Wageningen University, P.O. Box 338, Wageningen, 6700 AH, The Netherlands; 2 Centre for Infectious Disease Control Netherlands, National Institute for Public Health and the Environment, P.O. Box 1, Bilthoven, 3720 BA, The Netherlands; 3 Department of Veterinary Disease Biology, Faculty of Life Sciences, University of Copenhagen, Stigbøjlen 4, Frederiksberg C, 1870, Denmark; 4 Pig Health Department, Animal Health Service, P.O. Box 9, Deventer, 7400 AA, The Netherlands; 5 Operational Directorate of Bacterial Diseases, Veterinary and Agrochemical Research Centre, Groeselenberg 99, Bruxelles, 1180, Belgium; 6 Department of Pathology, Bacteriology and Avian diseases, Faculty of Veterinary Medicine, Ghent University, Salisburylaan 133, Merelbeke, B-9820, Belgium; 7 Department of Large Animal Sciences, Faculty of Life Sciences, University of Copenhagen, Stigbøjlen 4, Frederiksberg C, 1870, Denmark

**Keywords:** Methicillin resistant, Staphylococcus aureus, Transmission, Pigs, MRSA, Reproduction ratio

## Abstract

**Background:**

Since the detection of MRSA CC398 in pigs in 2004, it has emerged in livestock worldwide. MRSA CC398 has been found in people in contact with livestock and thus has become a public health issue. Data from a large-scale longitudinal study in two Danish and four Dutch pig herds were used to quantify MRSA CC398 transmission rates within pig herds and to identify factors affecting transmission between pigs.

**Results:**

Sows and their offspring were sampled at varying intervals during a production cycle. Overall MRSA prevalence of sows increased from 33% before farrowing to 77% before weaning. Overall MRSA prevalence of piglets was > 60% during the entire study period. The recurrent finding of MRSA in the majority of individuals indicates true colonization or might be the result of contamination. Transmission rates were estimated using a Susceptible-Infectious-Susceptible (*SIS*-)model, which resulted in values of the reproduction ratio *(R*_*0*_) varying from 0.24 to 8.08. Transmission rates were higher in pigs treated with tetracyclins and β-lactams compared to untreated pigs implying a selective advantage of MRSA CC398 when these antimicrobials are used. Furthermore, transmission rates were higher in pre-weaning pigs compared to post-weaning pigs which might be explained by an age-related susceptibility or the presence of the sow as a primary source of MRSA CC398. Finally, transmission rates increased with the relative increase of the infection pressure within the pen compared to the total infection pressure, implying that within-pen transmission is a more important route compared to between-pen transmission and transmission through environmental exposure.

**Conclusion:**

Our results indicate that MRSA CC398 is able to spread and persist in pig herds, resulting in an endemic situation. Transmission rates are affected by the use of selective antimicrobials and by the age of pigs.

## Background

In 2004, a distinct clone of methicillin resistant *Staphylococcus aureus* (MRSA CC398), referred to as livestock-associated (LA), was found in pigs and in people in contact with pigs [1]. Various observational studies have detected LA-MRSA in pig and other livestock herds worldwide, and risk factors for herds to be MRSA positive have been identified [2-7].

Antimicrobial resistant microorganisms in livestock become a public health issue when resistant organisms or resistance genes can transfer from livestock to humans. The role of animal populations in the transmission of microorganisms to humans is not only dependent on the possibility of transmission from animals to humans, but also on the possibility of transmission between animals.

The primary route of MRSA transmission between humans seems to be direct contact with individuals carrying MRSA [8,9]. However, environmental spread might be a substantially underestimated route for MRSA transmission in hospitals [10,11]. Similar mechanisms are likely for MRSA transmission between pigs. MRSA is not only isolated from pig mucosa and skin, but also from the herd environment [6,7,12], indicating that both direct and indirect transmission can occur. Little is known about MRSA transmission within pig herds and about the colonization dynamics of individual pigs over time. A single study in one pig herd assessed MRSA colonization in piglets over time and showed age-related differences in MRSA prevalence in young pigs (< 10 weeks) [13].

Transmission can be measured in longitudinal field studies and experiments, and can be expressed with the reproduction ratio (*R*_*0*_), which is an essential parameter in management of diseases. *R*_*0*_ is defined as the average number of secondary cases caused by one typical infectious individual during its entire infectious period in a completely susceptible population, and is often used as a quantitative measure of transmission [14,15]. *R*_*0*_ has a threshold value of 1; if *R*_*0*_ > 1, minor and major outbreaks can occur and an endemic situation can be established and maintained, whereas when *R*_*0*_ < 1 an infection does not spread and will not become endemic, *i.e.* the infection will fade out [15,16].

The objectives of this study were to quantify MRSA transmission rates and routes within pig herds and to identify factors affecting MRSA transmission between pigs. These objectives were obtained by a large-scale longitudinal study conducted in two Danish and four Dutch pig herds.

## Methods

### Selection of herds and sampling

Six farrow-to-finish herds, confirmed MRSA positive, were selected by convenience: two Danish herds (DK1, DK2) selected from a Danish pilot study [17], and four Dutch herds (NL1, NL2, NL3, NL4) selected from a cross-sectional prevalence study [7]. At each herd, one cohort of pregnant sows approaching delivery and placed in the same farrowing compartment was selected for sampling, except for herd NL4, where two cohorts of sows were included (NL4a and NL4b) with a time interval of three months.

Sows and their offspring were sampled six times during a production cycle (Table 1). Nasal swabs were taken from all pigs present at all sampling moments. Additionally, vaginal swabs were taken from the sows present at all moments, and rectal swabs were taken from new born piglets. At all sampling moments, four or five environmental wipes (Sodibox, s1 kit ringer solution, France) were taken from surfaces of each selected herd compartment.

**Table 1:** Sampling moment, compartment, time in life, age group and sample type per herd

**Moment**^**1)**^	**Compartment**	**Approximate time in life**	**Age group**	**Type of sample**	
				**Sows**	**Pigs**
1	Farrowing	1 week before farrowing	Sows	Nasal/vaginal	-
2	Farrowing	3 days after farrowing/birth	Sows/piglets	Nasal/vaginal	Nasal/rectal
3	Farrowing	3 wks after farrowing/birth	Sows/piglets	Nasal/vaginal	Nasal
4	Weaning^2)^	6 wks after birth	Pigs	-	Nasal
5	Weaning	10 wks after birth	Pigs	-	Nasal
6	Finishing^3)^	25 wks after birth	Pigs	-	Nasal

1) Each sampling moment, 4–5 environmental wipes were taken in each compartment.

2) Pigs were moved to the weaning section approximately 4 weeks after birth.

3) Pigs were moved to the finishing section approximately 11 weeks after birth.

In total, 63 sows and their offspring were included in the study. The number of sows and piglets at each sampling moment varied depending on the number of pigs born alive and/or on movement or death of pigs. After weaning, sows returned to the breeding compartment and were omitted from further sampling. The cohorts of pigs were monitored until slaughtering time. Some pigs were lost for follow up due to sorting and mixing of pigs to other compartments. In one Dutch herd (NL1), no samples were collected at the last two sampling moments, because the pigs were moved to another location.

The study protocol was in accordance with the Dutch Law on Animal Health and Welfare and discussed with the Animal Welfare Officer of Wageningen University. The distress was considered below the European injection criterium and therefore no further approval of the Animal Welfare Commission was needed. Informed consent was obtained from each participating farmer.

### Microbiological analysis

Samples were enriched using Mueller Hinton Broth with 6.5% NaCl (MHB+). Nasal, vaginal and rectal swabs were placed into 5–10 ml MHB+, environmental wipes into 100 ml MHB+. After 18 h of aerobic incubation at 37°C, a loop-full of MHB + was spread onto a chromogenic MRSA Brilliance agar (Oxoid, PO5196A, UK). One suspected colony per sample was confirmed to be MRSA CC398 by PCR [18,19].

### Data analysis

At each sampling moment, prevalences of positive individuals were calculated. An individual was considered positive if either one of the swabs (nasal, rectal or vaginal) tested positive for MRSA. The exact confidence intervals (exact 95% CI) for these prevalences were calculated based on the binomial probability function (PROC FREQ) [20].

A susceptible-infectious-susceptible (*SIS*) model [16] was used to describe the transmission of LA-MRSA within herds. For each moment, pigs were classified infectious (*I*) if either one of the swabs tested positive for MRSA. Pigs were classified susceptible (*S*) if all swabs tested negative for MRSA. New born piglets were assumed to be MRSA negative, thus susceptible, at birth. Three sources of MRSA for a susceptible pig were allocated in our study: i) infectious individuals, including sows, within the pen; ii) infectious individuals, including sows, within the compartment (but not in the same pen); and iii) the environment of the compartment. The term total infection pressure (*IP*) was introduced and defined as the sum of the proportion of infectious pigs (piglets + sows) within the pen (*IP* within the pen), the proportion of infectious pigs (piglets + sows) within the compartment, but not in the same pen (*IP* other pens), and the proportion of positive environmental wipes (*IP* environment).

**Figure 1:**
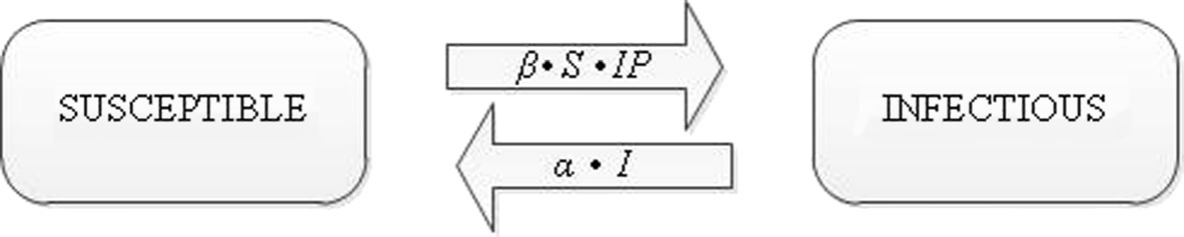
SIS-model used to quantify MRSA transmission rates.

The *SIS*-model can then be represented in Figure 1.

In this model, *β* is the transmission parameter, defined as the number of secondary cases (*C*) out of a number of susceptible individuals (*S*) caused by a certain infection pressure (*IP*) during each time interval between samplings (*Δt*). The number of new cases (*C*) per time interval (*Δt*) depends directly on *β*, *S* and *IP*; infectious individuals (*I*) become susceptible again at recovery rate *α*. *C, I, S* and *Δt* were determined per pen. Underlying assumptions were that within a pen (1) all pigs were randomly in contact, (2) susceptible and infectious individuals were homogenous groups, *i.e.* all individuals were equally susceptible or infectious, and (3) infectious individuals were equally infectious over time.

The probability for each piglet to become MRSA positive during a time period *Δt* depends on the transmission rate *β* and the infectiousness present in their surroundings which we defined as IP. The probability for each piglet to become MRSA positive is therefore equal to: $1-e^{-}{}^{\beta \cdot IP\cdot \Delta t}$

From this probability, it can be shown that the number of new cases (*C*) in a period *Δt* follows a binomial distribution with parameter $1-e^{-}{}^{\beta \cdot IP\cdot \Delta t}$and index *S*, the number of susceptible individuals (in our case MRSA negative animals) at the start of each time period (*Δt*). Consequently, the relation between the expected number of cases per unit of time (E(C)) and transmission rate *β*, *IP* and the number of susceptibles is as follows (see Velthuis *et al.* for a detailed explanation [21]):

$$\;E\left(C\right)=S\cdot \left(1-e^{-\beta \cdot \Delta t}\right)$$

Our data (summarized in Table 2) were statistically analysed with SAS® 9.1 using Generalized Linear Models (described by McCullagh and Nelder [22]; PROC GENMOD [20]) with a complementary log-log link function, the term log (*IP ·Δt*) as offset variable, *C* as the number of new cases, and *S* as the number of trials in the binomial process. Use of such generalized linear models using an offset as explanatory variable on infectious disease data is described by Becker [23] in more detail. The relation between the expected value (E) of a number of new cases out of a number of susceptibles during a time period *Δt* is presented in the following basic statistical model:

$$\;c\log\log\left(E\;C/S\right)=\log\left(\beta \right)+\log\left(IP\cdot \Delta t\right)$$

**Table 2:** Summarized model input per herd for piglets only used for estimation of MRSA transmission parameters

**Herd**	**Interval**^**1)**^	**Infectious (n/compartment)**	**Susceptible (n/compartment)**	**Cases (n/compartment)**	***Δt*(days)**	**Pens**^**2)**^**(n)**	**Risk ab**^**3)**^**(n pens)**	**p*IP*(range)**
NL1	I	0	199	85	4	16	9	0.00-0.47
	II	34	56	50	14	10	4	0.28-0.34
	III	92	11	11	21	5	5	0.33-0.33
	IV	-	-	-	-	-	-	-
	V	-	-	-	-	-	-	-
NL2	I	0	147	147	6	12	12	0.33-0.33
	II	-	-	-	-	-	-	-
	III	-	-	-	-	-	-	-
	IV	36	40	40	21	10	0	0.40-0.40
	V	-	-	-	-	-	-	-
NL3	I	0	90	86	5	8	8	0.28-0.34
	II	17	4	4	14	2	0	0.33-0.33
	III	42	1	1	21	1	0	0.33-0.33
	IV	-	-	-	-	-	-	-
	V	-	-	-	-	-	-	-
NL4a	I	0	68	3	2	6	0	0.00-0.91
	II	3	61	1	21	6	1	0.00-1.00
	III	2	62	31	13	6	0	0.23-0.70
	IV	23	30	23	21	5	0	0.33-0.47
	V	16	2	1	80	2	0	0.20-0.34
NL4b	I	0	79	1	4	6	0	0.00-1.00
	II	-	-	-	-	-	-	-
	III	0	67	14	24	6	0	0.00-0.51
	IV	14	53	7	15	6	0	0.30-0.66
	V	4	19	8	94	2	0	0.12-0.88
NL		283	989	513		109	39	0.00-1.00^4)^
DK1	I	0	57	57	3	5	0	0.31-0.31
	II	-	-	-	-	-	-	-
	III	-	-	-	-	-	-	-
	IV	-	-	-	-	-	-	-
	V	-	-	-	-	-	-	-
DK2	I	0	142	97	4	14	14	0.05-0.37
	II	27	41	34	18	7	0	0.23-0.32
	III	86	17	17	22	7	1	0.22-0.22
	IV	-	-	-	-	-	-	-
	V	-	-	-	-	-	-	-
DK		113	257	205		33	15	0.05-0.37^4)^

NL = Dutch; DK = Danish.

1) Interval I–V = time between subsequent sampling moments starting from birth.

2) Number of pens used in statistical analysis.

3) Number of pens where > 1 pig received treatment with risk antimicrobials, *i.e.* tetracyclins or β-lactams.

4) NL: mean = 0.32; SD = 0.21, DK: mean = 0.27; SD = 0.08.

– : No susceptibles left.

Exponentiation of the estimated parameter log (*β*) gives the transmission parameter *β*, that denotes the transmission per day. The reproduction ratio *R*_*0*_ can then be calculated by multiplying *β* with the length of the infectious period (1/*α*). We assumed 17.4 days to be the length of the infectious period. This was based on the average length of the infectious period observed in a transmission experiment [24] and on the interval of sampling in this study, which ranged from 4 to 94 days.

To the basic statistical model, more explanatory variables potentially affecting transmission were added. These variables were related to antimicrobial use, age and contribution of direct and indirect transmission. Tetracyclins and β-lactam antimicrobials were defined as risk antimicrobials (ab), as these antimicrobial classes select 100% for MRSA CC398 [25] and will potentially affect its transmission. If these antimicrobials were applied on >1 pig within a pen during a time interval, then variable ‘ab’ was defined as yes, otherwise no. Age of pigs was introduced as variable, because an age-effect or presence of the sow in the farrowing compartment might affect transmission of MRSA compared to transmission in post-weaning pigs. When pigs were located in the farrowing compartment, ‘age’ was defined as pre-weaning, and after weaning when located in the weaning or finishing compartment as post-weaning. To quantify the relative effect of transmission through direct contact with pen mates compared to the total transmission through direct and indirect contact, a continuous explanatory variable was introduced. This variable (pIP) was calculated as *IP* within the pen divided by the total *IP*. A multivariable model, including the explanatory variables described above, is presented as follows:

$$\begin{array}{ccc} \;c\log\log\left(E\;C/S\right) & \;= & \log\left(\beta \right)+log\left(IP^{*}\Delta t\right)+\log\left(ab\right)\\ & \;+ & \log\left(age\right)+\log\left(pIP\right) \end{array}$$

**Table 3:** Models used to quantify transmission of MRSA within pig herds

**Model**	**Transmission parameter estimation**^**1)**^	**Used data**
Basic	*TP*_*1*_ = exp (log *β*_*0*_*)*	Denmark / Netherlands
Bivariable	*TP*_*2*_ = exp (log *β*_*0*_ + log *β*_1_(ab) + log *β*_2_(pIP))	Denmark / Netherlands
Multivariable	*TP*_*3*_ = exp (log *β*_*0*_ + log *β*_1_(ab) + log *β*_*2*_(pIP) + log *β*_3_(age))	Netherlands

1) In all models log (*IP·Δt*) was used as offset variable.

ab: use of tetracyclins or β-lactams (yes/no); age: age of pigs (pre-weaning/post-weaning); pIP: infection pressure within the pen divided by total infection pressure (continuous variable).

Data from Denmark and The Netherlands were analysed separately and in combination. First, analysis was done without explanatory variables to estimate a basic transmission parameter (Table 3). Secondly, analysis including explanatory variables was done. Due to very high prevalences in new born piglets in both Danish herds, the number of susceptibles was very low after sampling moment 2, leaving no or very few cases to occur at post-weaning age. Analysis was, therefore, done on data from pre-weaning pigs only, implying that the explanatory variable ‘age’ could not be included in this analysis (Table 3). Finally, multivariable analysis was performed on Dutch data only from all pigs.

In bi- and multivariable analysis two-way interactions between variables were tested for significance and removed if *P >* 0.05. To estimate solely the herd effect, *i.e.* without explanatory variables, herd was included in the basic model as random effect using an exchangeable covariance structure (PROC GENMOD) [22]. Herd effect for Dutch data only accounted for 0.06% of non-explained variance, and was therefore not included in the statistical models. Herd effect for Danish data could not be estimated.

## Results

### MRSA prevalence

Overall MRSA prevalence of sows increased from 33.3% (exact 95% CI: 22.0-46.3%) before farrowing (moment 1) to 58.8% (exact 95% CI: 46.2-70.6%) after farrowing (moment 2) and to 77.3% (exact 95% CI: 65.3-86.7%) ~ 3 weeks later, just before weaning (moment 3). All sows in herd NL4 tested MRSA negative, whereas all sows in herd DK1 tested MRSA positive at all sampling moments (Table 4).

**Table 4:** Numbers and percentages of MRSA positive sows and environmental wipes

	**Moment 1**			**Moment 2**			**Moment 3**		
	**Wipes**	**Sows**		**Wipes**	**Sows**		**Wipes**	**Sows**	
**Herd**	**N pos/n total**	**N**	**% Pos**	**N pos/n total**	**N**	**% Pos**	**N pos/n total**	**N**	**% Pos**
NL1	1/4	16	6.3	3/4	16	25.0	4/4	16	93.8
NL2	4/4	12	33.3	4/4	12	100.0	4/4	12	100.0
NL3	3/4	8	50.0	4/4	8	62.5	4/4	8	100.0
NL4a	1/4	6	0.0	0/4	6	0.0	0/4	4	0.0
NL4b	0/4	6	0.0	0/4	6	0.0	0/4	6	0.0
NL	9/20	48	18.8	11/20	48	43.8	12/20	46	76.1
DK1	5/5	5	100.0	5/5	6	100.0	10/10	6	100.0
DK2	5/5	10	70.0	4/5	14	92.9	5/5	14	71.4
DK	10/10	15	80.0	9/10	20	95.0	15/15	20	80.0
NL + DK	19/30	63	33.3	20/30	68	58.8	27/35	66	77.3

NL = Dutch; DK = Danish.

Sows were classified MRSA positive if either one of the swabs (nasal/vaginal) tested positive; from all MRSA positive samplings of sows (*n* = 112), 58% was classified positive based on both swabs, 39% on a positive nasal swab and 3% on a positive vaginal swab.

Pre-weaning, overall MRSA prevalence of pigs increased from 60.9% (exact 95% CI: 57.4-64.3%) in new born piglets (moment 2) to 77.8% (exact 95% CI: 74.6-80.7%) ~ 3 weeks later, just before weaning (moment 3). Post-weaning, MRSA prevalence of pigs was 79.6% (exact 95% CI: 76.2-82.8%) at moment 4 and 86.6% (exact 95% CI: 83.2-89.5%) at moment 5. In the finishing compartment, just before slaughter, MRSA prevalence was 69.6% (exact 95% CI: 64.9-74.1 (Table 5).

**Table 5:** Numbers and percentages of MRSA positive pigs and environmental wipes

	**Moment 2**		**Moment 3**		**Moment 4**			**Moment 5**			**Moment 6**		
	**Pigs**		**Pigs**		**Wipes**	**Pigs**		**Wipes**	**Pigs**		**Wipes**	**Pigs**	
**Herd**	**N**	**% Pos**	**N**	**% Pos**	**N pos/N total**	**N**	**% Pos**	**N pos/n total**	**N**	**% Pos**	**N pos/n total**	**N**	**% Pos**
NL1	199	42.7	186	93.0	4/4	125	100.0	-	-	-	-	-	-
NL2	147	100.0	146	99.3	0/4	93	55.9	2/4	92	100.0	4/4	80	86.3
NL3	90	95.6	90	98.9	4/4	87	100.0	4/4	86	100.0	4/4	82	63.4
NL4a	68	4.4	64	3.1	0/4	65	52.3	1/4	64	89.1	3/4	62	64.5
NL4b	79	1.3	70	0.0	1/4	67	20.9	0/4	68	16.2	0/4	68	13.2
NL	583	55.2	556	73.6	9/20	437	71.4	7/16	310	79.4	11/16	292	58.2
DK1	57	100.0	48	100.0	10/10	48	100.0	7/10	45	100.0	5/5	46	100.0
DK2	142	68.3	133	87.2	10/10	129	100.0	15/15	123	100.0	10/10	67	98.5
DK	199	77.4	181	90.6	20/20	177	100.0	22/25	168	100.0	15/15	113	99.1
NL + DK	782	60.9	737	77.8	29/40	614	79.6	29/41	478	86.6	26/31	405	69.6

NL = Dutch; DK = Danish.

Pigs were classified MRSA positive if either one of the swabs (nasal/rectal) tested positive; from all MRSA positive samplings of pigs (*n* = 476), 67% was classified positive based on both swabs, 31% on a positive nasal swab and 2% on a positive rectal swab.

New born piglets (moment 2) in Dutch herds were more often MRSA positive (*P* < 0.0001) if their dam was MRSA positive before farrowing compared to MRSA negative dams. MRSA prevalence in pigs from positive dams was 84% *versus* 48% from negative dams. In Danish herds, MRSA prevalence in pigs from positive dams was 78% *versus* 73% from negative dams. Combining data from all herds, 81% of new born pigs from MRSA positive sows were positive after birth, whereas 50% of pigs from MRSA negative sows were positive at that time (*P* < 0.0001).

The number of MRSA positive environmental wipes varied largely between herds and sampling moments, from no positive wipes to all wipes positive (Table 4 and 5).

### Transmission quantification

Table 2 shows model input summarized per herd per sampling interval, which was used for MRSA transmission quantification. Basic *R*_*0*_-values including data from all pigs in the basic model, were 1.11 (95% CI: 1.00-1.22) for Dutch pigs and 1.88 (95% CI: 1.59-2.22) for Danish pigs. Including data from pre-weaning pigs only, resulted in a slightly higher *R*_*0*_-value for Dutch pigs (*R*_*0*_ = 1.96; 95% CI: 1.74-2.20), whereas the *R*_*0*_-value for Danish pigs did not change (*R*_*0*_ = 1.88; 95% CI: 1.59-2.22). Analysis on data from both countries resulted in intermediate *R*_*0*_-values (Table 6).

**Table 6:** **MRSA transmission per day and *R***_***0***_**-values**

	**Pre-weaning pigs only**				**All pigs**			
**Country**	***C/S***^**2)**^	***TP***_***1***_	***R***_***0***_	**95% CI**	***C/S***^**2)**^	***TP***_***1***_	***R***_***0***_	**95% CI**
NL	377/704	0.112	1.96	1.74 - 2.20	513/989	0.064	1.11	1.00 - 1.22
DK	188/240	0.108	1.88	1.59 - 2.22	205/257	0.108	1.88	1.59 - 2.22
NL + DK	565/944	0.111	1.93	1.75 - 2.13	718/1246	0.071	1.24	1.14 - 1.35

Legend: This table shows the transmission per day from the basic model (*TP*_*1*_) and *R*_*0*_-value^1)^ with its 95% confidence interval for Dutch (NL) and Danish (DK) herds separately and all herds (NL + DK) for pre-weaning pigs only and for all pigs.

1) To calculate *R*_*0*_, *TP*_*1*_ was multiplied with 17.4 days (= length of infectious period).

2) Number of MRSA cases (*C*) from total number of susceptibles (*S*) used in analysis.

Bivariable analysis of data from pre-weaning pigs only, showed effects of both variables: use of risk antimicrobials (ab: *P* < 0.0001 for Dutch data and *P* = 0.10 for Danish data) and the relative proportion of *IP* within the pen compared to the total *IP* (pIP: *P* < 0.0001 for Danish and Dutch data), and no significant interaction effect (*P >* 0.05). The effect of pIP was very large, especially in Danish herds. For Danish pigs, *R*_*0*_ was 0.02 (95% CI: 0.00-0.07) without use of risk antimicrobials and pIP at its minimum (*i.e.* 0), and 0.03 (95% CI: 0.00-0.16) with use of risk antimicrobials and pIP at its minimum. When pIP was at its maximum, *R*_*0*_-values were infinitely large both without and with use of risk antimicrobials (*R*_*0*_ = 121076 (95% CI: 307.31-4.77E + 07) and 1760410 (95%CI: 287.92-1.08E + 08), respectively). For Dutch pigs, *R*_*0*_ was 0.62 (95% CI: 0.45-0.84) without use of risk antimicrobials and pIP at its minimum, and 1.54 (95% CI: 0.84-2.83) with use of risk antimicrobials and pIP at its minimum. When pIP was at its maximum, *R*_*0*_-values were 3.28 (95% CI: 1.43-7.49), and 8.17 (95%CI: 2.65-25.22) for without and with use of risk antimicrobials, respectively (Table 7). Analysis on data from both countries resulted in similar results, with the lowest *R*_*0*_ when risk antimicrobials were not used and pIP at its minimum (*R*_*0*_ = 0.68; 95% CI: 0.54-0.85), and the highest *R*_*0*_ when risk antimicrobials were used and pIP at its maximum (*R*_*0*_ = 10.50; 95% CI: 4.31-25.59). Average pIP was 0.32 (SD = 0.21) for Dutch herds and 0.27 (SD = 0.08) for Danish herds (Table 2).

**Table 7:** **MRSA transmission parameter and *R***_***0***_**-values**

		**No risk antimicrobials; pIP = 0**			**Risk antimicrobials; pIP = 0**			**No risk antimicrobials; pIP = 1**			**Risk antimicrobials; pIP = 1**		
**Country**	***C/S***^**4)**^	***TP***_***2***_	***R***_***0***_	**95% CI**	***TP***_***2***_	***R***_***0***_	**95% CI**	***TP***_***2***_	***R***_***0***_	**95% CI**	***TP***_***2***_	***R***_***0***_	**95% CI**
NL	377/704	0.035	0.62	0.45 - 0.84	0.089	1.54	0.84 - 2.83	0.188	3.28	1.43 - 7.49	0.470	8.17	2.65 - 25.22
DK	188/240	0.001	0.02	0.00 - 0.07	0.002	0.03	0.00 – 0.16	6958	121076	307.31 - ∞	10117	176041	287.92 - ∞
NL + DK	565/944	0.039	0.68	0.54 - 0.85	0.076	1.32	0.84 - 2.06	0.309	5.38	2.74–10.56	0.603	10.50	4.31 - 25.59

Legend: this table shows the transmission per day resulting from bivariable analysis with use of risk antimcrobials^1)^ and the relative proportion of IP compared to the total IP (pIP) included in the model^2)^ (*TP*_*2*_) and *R*_*0*_-value^3)^ with its 95% confidence interval for Dutch (NL) and Danish (DK) farms separately and all farms combined (NL + DK) for pre-weaning pigs only.

1) Risk antimicrobials (tetracyclins and β-lactams) in > 1 pig per pen.

2) Interaction between variables was not significant (*P* > 0.05).

3) To calculate *R*_*0*_, *TP*_*2*_was multiplied with 17.4 days (= length of infectious period).

4) Number of MRSA cases (*C*) from total number of susceptibles (*S*) used in analysis.

**Figure 2:**
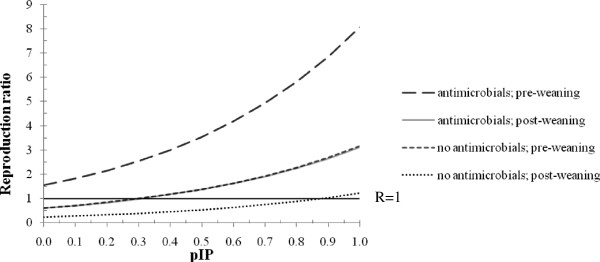
**Reproduction ratio for MRSA in pigs related to antimicrobial use, age and infection pressure.** Reproduction ratio related to use of tetracyclins and β-lactam antimicrobials (yes, no), age of pigs (pre-weaning, post-weaning) and relative proportion of the infection pressure within the pen compared to the total infection pressure (pIP); based on 513 MRSA cases (*C*) from 989 susceptibles (*S*) from 4 herds. Note: lines for antimicrobials; post-weaning pigs and no antimicrobials; pre-weaning pigs are overlapping, thus difficult to distinguish.

Multivariable analysis of all Dutch data showed significant effects of all three variables, ab, age and pIP (*P* < 0.0001); interaction effects were not significant (*P* > 0.05). Figure 2 shows the effect of ab and age on *R*_*0*_ for pIP-values between 0 and 1. *R*_*0*_ was lowest at 0.24 (95% CI: 0.18-0.31) when no risk antimicrobials were used in post-weaning pigs and pIP = 0. *R*_*0*_ increased to 0.60 (95% CI: 0.34-1.06) when risk antimicrobials were used in post-weaning pigs and a minimal pIP. *R*_*0*_ was above 1, though not significant (*R*_*0*_ = 1.56; 95% CI: 0.65-3.77), when risk antimicrobials were used in pre-weaning pigs and a minimal pIP, and below 1, though not significant, (*R*_*0*_ = 0.61; 95% CI: 0.34-1.10) when risk antimicrobials were used in post-weaning pigs and a minimal pIP. *R*_*0*_ increases with increasing pIP. Given a maximal pIP of 1, *R*_*0*_ was 1.22 (95% CI: 0.60-2.48) without using risk antimicrobials in post-weaning pigs and significantly above 1, *i.e.* 3.12 (95% CI: 1.15-8.46) when risk antimicrobials were used in this age-group. In pre-weaning pigs, *R*_*0*_-values were significantly above 1; *R*_*0*_ was 3.16 (95% CI: 1.14-8.82) when no risk antimicrobials were used, and highest with use of risk antimicrobials (*R*_*0*_ = 8.08; 95% CI: 2.17-30.12).

## Discussion

MRSA prevalences in different age groups and transmission rates between pigs were assessed longitudinally in six herds in two European countries. Sow prevalences varied widely between herds and over time. This might be explained by differences in management practices. In The Netherlands, an all in – all out system is applied in the farrowing compartment, *i.e.* a cohort of sows due to farrow is placed into a ‘clean’ farrowing compartment, whereas in Denmark a continuous system is practiced. Except for one Dutch herd, prevalence in sows increased in the Dutch herds during time in the farrowing compartment, which might be explained by a build-up of bacterial load, *i.e.* the infection pressure, during time spent in this compartment. However, given the small sample of herds studied in each country, these differences could also be due to specific management factors at the herds under study and not necessarily reflect differences between the two countries.

Prevalences in new born piglets varied from 1 to 100%. A similar explanation as given for differences in prevalences in sows might be applicable here. In one Dutch herd, prevalences in pre-weaning pigs remained low (< 5%). A rapid increase in prevalence in this herd was seen after weaning, despite the fact that no risk antimicrobials were used during that time interval and that no positive dust samples were found at the first sampling moment in the weaning compartment. In a longitudinal study on an antimicrobial-free Canadian pig farm, a similar increase in MRSA prevalence was observed around the time of weaning [13]. Co-mingling of MRSA positive and negative pigs, transmission through human handling during weaning, increased susceptibility due to stress or related to age, or a combination of these factors, might be responsible for the rapid increase in MRSA prevalence after weaning.

The recurrent finding of MRSA in the majority of sampled individuals either indicates true colonization or might be the result of contamination. To distinguish between true colonization and contamination, MRSA positive pigs should be placed in a clean environment individually for a longer time.

We used a *SIS*-model to describe the transmission of MRSA within herds, assuming that infectious pigs stop shedding after a while and become susceptible again. This assumption was based on the fact that most humans are intermittent carriers [26] and on data from a former experimental study [24].

The basic reproduction ratio, based on a *SIS*-model without explanatory variables, was significantly above one, indicating a high probability of transmission and persistence within a pig herd [27]. The reproduction ratios were calculated by multiplying estimated transmission parameters with the length of the infectious period. An infectious period is only valid for infectious individuals. Because MRSA can survive outside the host for long periods [28], the environment might also be a source of MRSA. How the contamination of the environment reflects MRSA prevalence of pigs in this environment, how long LA-MRSA persists in the environment, and how environmental contamination affects transmission, is unknown. Our method for quantification of the reproduction ratio might, therefore, be less applicable in situations where no or very few pigs are MRSA positive within a compartment, and where MRSA is present in the environment. For our calculations we used 17.4 days as the length of the infectious period, whereas the final observational time interval, *i.e.* the period in the finishing pig compartment, was much longer (> 10 weeks). During this period, more than one infection might have occurred in one individual, whereas our method only counts one. This implies a potential underestimation of transmission rates. Based on the prevalence in pigs just before slaughter (70%), the reproduction ratio can be estimated using *R*_*0*_ = 100/(100-prevalence) [27], resulting in 3.27. This is indeed higher than the estimated basic reproduction ratio based on data from all pigs in both countries (*R*_*0*_ = 1.24), indicating an underestimation, however, it is similar to the estimated reproduction ratio from a transmission experiment [24].

Transmission rates were higher when tetracyclins and β-lactams were used which might be explained by a selective advantage of MRSA CC398 as compared to susceptible strains present in the nasal microbiota when these antimicrobials are used. An experimental study investigating the effects of zinc and tetracycline on MRSA counts in nasal samples of pigs, showed higher counts in treated animals than in untreated animals, which seems to confirm a selective advantage of MRSA CC398 caused by both compounds [29]. The effect of zinc could not be assessed in our study as only Danish herds applied zinc in the weaning compartment where all pigs were already MRSA positive before entering.

The proportion of susceptibles and therefore, potentially new cases, was relatively high among pre-weaning pigs compared to post-weaning pigs; we actually assumed all new born piglets to be MRSA negative, and thus susceptible, before birth. MRSA prevalence in post-weaning pigs was much higher, leaving fewer susceptibles to become a case and thus estimation of transmission rates was based on less information in this age-group. Thus the power of the comparison is lower. Nevertheless, the available data indicated that transmission rates in pre-weaning pigs are significantly higher than in post-weaning pigs. This might be explained by the presence of the sow, which might be a primary source of MRSA for the new born piglets. An association between MRSA status of the sow prior to farrowing and that of the offspring just after birth was shown in our study, and was found in a longitudinal study on a Canadian pig farm as well [13]. Perinatal transmission of MRSA CC398 from sow to pigs has been demonstrated under controlled experimental conditions [30]. New born piglets might be more susceptible to acquisition of MRSA and other infectious agents due to their immature mucosal immune system and the greater impact of antimicrobials on their unbalanced microbiota [31,32].

The increased transmission rates observed in correspondence with the relative increase of the infection pressure within the pen implies that transmission through direct contact with pen mates is an important transmission route. More quantitative information on transmission rates within and between pens and the role of environmental contamination can be obtained by transmission experiments [33].

Since only 4 to 6 farrow-to-finish herds were included in the estimation of transmission rates, including some herds with very high prevalences leaving just a few trials for parameter estimation, the results might not be representative for the international pig herd population. Moreover, the observed association between explanatory variables and *R*_*0*_, *e.g.* antimicrobial use, might be confounded by other effects. Although herd effect accounted for only 0.06% of the variance in the multivariable analysis, it was not possible to distinguish the herd effect from an unconfounded estimate of the exposure effects. Prudence is therefore called for in drawing conclusions from these associations.

## Conclusion

In conclusion, introduction of MRSA in a fully susceptible population most probably leads to transmission and an endemic situation where direct contact between animals is the most important route of transmission. Control programs should therefore focus on (1) prevention of introduction into a herd, and (2) prevention of transmission within a herd, *e.g.* by prudent antimicrobial use, all-in all-out procedures and hygiene barriers between age groups.

## Competing interests

The authors declare that they have no competing interests.

## Authors' contributions

MJ, EG, AG, LG, PB, JPN, PW and EB contributed to the design of the longitudinal field study. EB, PB, PW and CEG coordinated and performed the studies. EG, NV, MJ and EB analysed the data. EB, EG and CEG drafted the manuscript. All authors read, revised and approved the final manuscript.

## Authors’ information

EB’s current position is at Department of Infectious Diseases and Immunology, Faculty of Veterinary Medicine, Utrecht University, The Netherlands
